# Fatty Acid Composition, Phytochemistry, Antioxidant Activity on Seed Coat and Kernel of *Paeonia ostii* from Main Geographic Production Areas

**DOI:** 10.3390/foods9010030

**Published:** 2019-12-28

**Authors:** Li-Ping Peng, Si-Qi Men, Zheng-An Liu, Ning-Ning Tong, Muhammad Imran, Qing-Yan Shu

**Affiliations:** 1 Key Laboratory of Plant Resources and Beijing Botanical Garden, Institute of Botany, Chinese Academy of Sciences, Beijing 100093, China; pengliping@ibcas.ac.cn (L.-P.P.); 18270839987@163.com (S.-Q.M.); liuzhengan@ibcas.ac.cn (Z.-A.L.); tongnn@ibcas.ac.cn (N.-N.T.); 2 Yanqi Lake Campus, University of Chinese Academy of Sciences, Beijing 100049, China; 3 State Key Laboratory of Plant Cell and Chromosome Engineering, Institute of Genetics and Developmental Biology, Chinese Academy of Sciences, Beijing 100101, China; imran_m1303@yahoo.com

**Keywords:** unsaturated fatty acids, vegetable oil, antioxidant activity, *P. ostii*, seed quality

## Abstract

*Paeonia ostii* is an important woody oil plant cultivated in China on a large scale. Its seed oil is enriched with unsaturated fatty acids and a high content of alpha-linolenic acid (ALA), which are beneficial to human health. The aim of this research is to determine the qualitative traits characteristic of *P. ostii* seed from various production areas in China. In this study, seed quality traits were evaluated on the basis of proximate composition, content of fatty acids, tocopherol, secondary metabolites, and the antioxidant activity of seed coat (PSC) and kernel (PSK). A high content of total fatty acids (298.89–399.34 mg g^−1^), crude protein (16.91%–22.73%), and total tocopherols (167.83–276.70 μg g^−1^) were obtained from PSK. Significant differences were found in the content of palmitic acids (11.31–14.27 mg g^−1^), stearic acids (2.42–4.24 mg g^−1^), oleic acids (111.25–157.63 mg g^−1^), linoleic acids (54.39–83.59 mg g^−1^), and ALA (99.85–144.71 mg g^−1^) in the 11 main production areas. Eight and seventeen compounds were detected in PSC and PSK, respectively. A significantly higher content of total phenols was observed in PSC (139.49 mg g^−1^) compared with PSK (3.04 mg g^−1^), which was positively related to antioxidant activity. This study indicates that seeds of *P. ostii* would be a good source of valuable oil and provides a basis for seed quality evaluation for the production of edible oil and potential ALA supplements from the promising woody oil plant.

## 1. Introduction

Plant seeds are a source of food, food ingredients, and supplements for human dietary needs in daily life [1]. Most of the seeds produce proteins, carbohydrates, numerous secondary metabolites, and can be used for edible oils [2,3]. Edible oils, such as olive oil, corn oil, and soybean oil are used for human diets and contain lipids in the form of fatty acids (FAs). Among FAs, α-linolenic acid (ALA), a kind of unsaturated fatty acid (UFAs), is an essential human dietary nutrient with health promoting effects that cannot be synthesized independently within the body [4]. Seeds from various plant species were detected with high levels of ALA, including *Ocimum basilicum* (50.10%–67.61%) [5,6], *Lasiococca comberi* (65.30%) [7], *Linum usitatissimum* (46.33%–55.47%) [8], *Eucommia ulmoides* (56.51%) [9], and *Paeonia suffruticosa* (21%–54%) [10]. ALA is the most important n-3 fatty acid from vegetables from a dietary perspective. However, in traditional vegetable oils, such as soybean (8.95%), canola (9.74%), corn germ (0.55%), sesame (0.32%), and olive (0.57%), n-3 fatty acids represent less than 10%, and hence do not meet human dietary needs [11]. Therefore, with improvements in living standards and the pursuit of healthy diets, the development of special edible oils rich in ALA from oil seed plants has become a worldwide demand. 

*Paeonia ostii* (Paeoniaceae, *Paeonia*, Sect. *Mutan* DC) is an emerging woody oil crop that originates in China, and is one of the most important plants, traditionally grown for the root bark that is used as an antispasmodic medicine throughout Asia [12]. It has been listed among the new promising woody crops for oil production in temperate regions due to its wide adaptability, low input requirement, and other advantages [13]. The legume of *P. ostii* has a star-shaped fruit, which contains dark oval seeds. In the industry, shelled seeds are used for oil production through cold pressed or subcritical extraction. Its seeds are an important source of edible oil for humans and possess economic potential due to their high amount of oil content (20%) and high yield (3.75 t/ha) [14]. However, it was long neglected for food industrial purposes until its seed oil was authenticated as a new food resource by the Ministry of Health of China in 2011 [15]. Its seed oil has been considered as a valuable vegetable oil and is enriched in UFAs (>90%) and ALA (40%) [10]. Currently, it is regarded as a rare source of ALA supplementation, particularly in comparison to commonly consumed vegetable oil, and has been widely planted in large numbers as a woody oil crop throughout China. However, in comparison to industrial needs, seeds used as raw material for edible oil must meet a high-quality standard. Therefore, it is necessary to conduct a comprehensive quality evaluation of seeds from different cultivated areas of China.

Chemical composition and nutritional evaluation of seeds and seed oils has been an important research field in the past few years, which has been focused on FA composition, antioxidant activities, protein, antinutrient, squalene, phytosterols, moisture, tocotrienol, and mineral contents in *Dalbergia odorifera* [16], *Acacia saligna* [17], *Salvia hispanica* [18], *Camellia oleifera* [19], and *Phoenix dactylifera* L. [20]. Previous studies have focused on systematic assessments of fatty acids of seeds from 60 *Paeonia* cultivars, which suggest that these detected cultivars could be good candidates as an oil resource for practical oil production [10]. More recently, attention has been focused on the analysis of FA composition in *Paeonia* plants [10,21], but few studies have assessed the chemical characterization and bioactive compounds.

In this study, the main purpose is to evaluate the proximate composition, main nutritional component, secondary metabolites, and antioxidant activity of *P. ostii* seed kernels (PSK) and seed coat (PSC) from 11 main production areas. This study can provide important standards for evaluating the seed quality of *P. ostii* to be used as a valuable source of vegetable oil, and can potentially identify interesting components for the processing and development of functional foods with high contents of ALA, crude protein, or tocopherol.

## 2. Materials and Methods 

### 2.1. Collection of Samples

**Figure 1:**
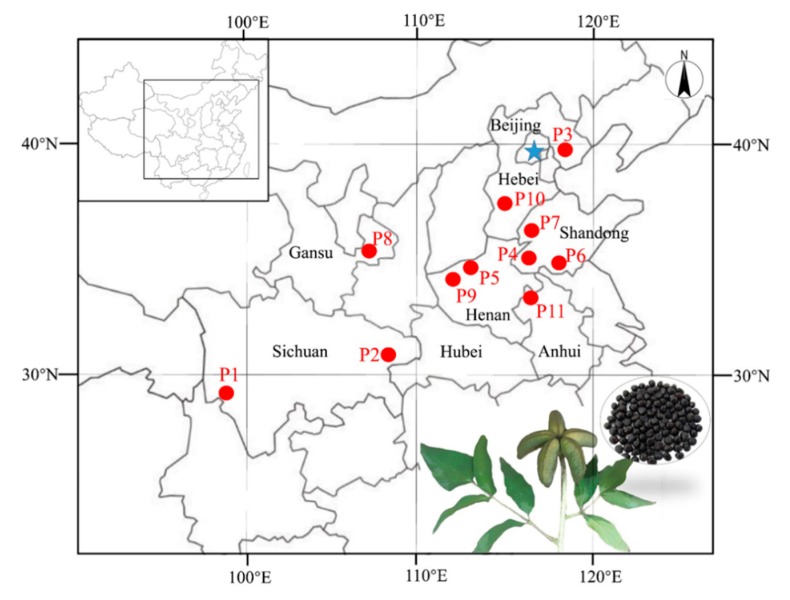
Location of the *P. ostii* seed collected from 11 production areas.

Mature seeds of *P. ostii* were collected in 2017 from eleven different production areas (Figure 1 and Table S1). The PSK and PSC from each location were separately grounded using a high-speed multi-purpose disintegrator (Zhejiang, China) before using them in further analyses.

### 2.2. Chemicals and Reagents

Fatty acid methyl esters (FAMEs) mixtures with 37-components and six FAMEs standard mixtures including palmitic acid (C16: 0, PA), methyl heptadecanoate (C17: 0), methyl stearate (C18: 0, stearic acid (SA)), methyl oleate (C18: 1∆9c, oleic acid (OA)), methyl linoleate (C18: 2∆9c, 12c, linolenic acid (LA)), ALA (C18: 3∆9c, 12c, 15c), and α-, β-, γ- and δ tocopherols standard were purchased from Sigma-Aldrich (St. Louis, MO, USA). Among them, methyl heptadecanoate was used as an internal standard (IS). Standard compounds of gallic acid and paeoniflorin were purchased from ANPEL Laboratory Technologies Inc. (Shanghai, China). All standards and stock solutions were kept in dark at 4 °C, while ALA was kept at −20 °C.

### 2.3. Seed Proximate Composition Analysis

The 100-seed weight, moisture content, and neatness were investigated according to Chinese standards of GB/T 3543.7-1995, GB/T 3543.6-1995, and GB/T 3543.3-1995. Crude protein content was determined by the micro-Kjeldhal method [22]. The seed coat rate was calculated by the following equation:

The seed coat rate (%) = (the weight of the seed coat (g))/(the weight of the whole seed (g)) × 100

### 2.4. Fatty Acid Composition and Content Analysis 

The seed lipids were extracted, and FAMEs analyses were prepared according to the procedures described previously [23]. Fatty acid analysis was performed using a gas chromatograph-mass spectrometer (GC-MS, GC6890N/MS5973, Aglilent Technoloties, Willmington, DE, USA) equipped with a flame-ionization detector (FID) and a capillary column (HP-88; 30 m × 0.25 mm, film thickness: 0.20 μm). The column temperature was maintained at 100 °C for 2 min, then elevated to 230 °C for 5 min at a rate of 15 °C/min. Ultra-high purity helium was used as the carrier gas at a flow rate of 1.0 mL/min. The injector temperature was set at 230 °C for split injection at a split ratio of 10:1. Identification of the peaks was achieved by retention time and comparing them with external standards analyzed under the same conditions.

### 2.5. Determination of the Components and Content of Tocopherol

The occurrence and content of four components of tocopherol (α-, β-, γ-, and δ tocopherols) in seeds of *P. ostii* was investigated according to GB 5009.82-2016 with modifications. Approximately 0.3 g of the sample and 0.05 g of amylase, together with 1.0 mL of ultrapure water, were added to a 10 mL glass tube at 60 °C for 45 min. After this, 1.5 mL of 100% ethanol containing 0.01 g of butylated hydroxytoluene (BHT), 0.1 g of ascorbic acid, and 1.5 mL of potassium hydroxide were added to the enzymatic hydrolysate and then kept at 80 °C for 60 min. Then, 2.5 mL of the extraction solvent mixture (petroleum ether:ethyl ether, 1:1) was added and mixed for 5 min and then followed by centrifugation. The ether layer was transferred into a tube, washed with distilled water at least 3 times until it had a slightly less than neutral pH value, and then the water phase was removed and the ether layer evaporated under nitrogen gas flow.

For the analysis, the ether layer was recovered in 1.0 mL of high-performance liquid chromatography (HPLC) grade methanol and filtered using a 0.22 μm membrane; 50 μL of the liquid was then injected into the chromatographic column for analysis. HPLC program: Acetonitrile-water (95:5) was applied for gradient elution for 40 min at a flow rate of 1.0 mL/min with the column temperature at 25 °C. Four tocopherols (α-, β-, γ- and δ-tocopherol) were used as external standards to generated calibration curves of peak area versus concentration.

### 2.6. Extraction and Assay of Phytochemical Compounds and HPLC-MS Analysis

Approximately 0.2 g of ground seed coat or kernel and 1.5 mL of extract solution (methanol:water = 70:30, *v*/*v*) were added into a 5 mL centrifuge tube and placed in an ultrasonic cleaner at 20 °C for 20 min. The supernatant was then transferred into another tube after centrifugation. The above procedure was repeated twice. The extract was filtered through a membrane (0.22 μm) and stored at −40 °C for further analysis.

For further HPLC analysis, the following solvent and gradient were used: A, 2% aqueous formic acid (*v*/*v*); B, 0.2% formic acid in acetonitrile (*v*/*v*); constant gradient from 5% to 64% of B within 80 min; a flow rate of 0.6 mL/min; and 10 μL of extract solution, injected for detection. The column temperature was maintained at 30 °C. DAD data were recorded at 280 nm. 

An Agilent 6520 Accurate-Mass Q-TOF LC/MS was used for the qualitative analysis of the compounds. The electrospray ionization mass spectrometry method was applied with positive or negative ion modes. The scanning range (*m*/*z*) was 100–1200 u, the sprayer pressure was 35 psi, and the capillary voltage was 3500 V. Dry gas at 350 °C was carried at a flow rate of 12 L/min. The data were analyzed by Masshunter Qualitative Analysis Software B. 04. 00. 

### 2.7. Total Phenolic Content and Antioxidant Activity Analysis

The total phenol content of the extracts was measured using the Folin-Ciocalteu (FC) reagent, as described by Li et al. [23], with some modifications. Quantities of 100 μL of sample solution, 2500 μL of ddH_2_O, and 100 μL of Folin-Ciocalteu reagent were added and mixed for 5 min, and then 300 μL of 20% Na_2_CO_3_ solution was added. The mixture was shaken and incubated at 37 °C in the dark for 2 h until the reaction reached a plateau. The absorbance was recorded at 750 nm using a spectrophotometer.

The antioxidant capacities of PSK and PSC were assessed by total phenol, 1,1-diphenyl-2-picrylhydrazyl (DPPH), 2,2′-azinobis-(3-ethylbenzothiazoline-6-sulfonicacid) (ABTS) and ferric reducing antioxidant power (FRAP) assays, which was established in our lab and described by Li et al. [23]. The identification of the radical scavenging activities and total phenolic content of samples was achieved using gallic acid as the standard [23,24]. 

### 2.8. Antioxidant Potency Composite (APC) Index Analysis

The APC index was used to evaluate the overall antioxidant activity of PSK and PSC [25]. The APC index was calculated by the followed formula:

APC index (%) = ((DPPH value)/(The maximum DPPH value) + (ABTS value)/(The maximum ABTS value) + (FRAP value)/(The maximum FRAP value))/3 × 100

### 2.9. Statistical Analysis

All of the chemical analyses were carried out in triplicate and values are expressed as the mean ± SD. Statistical significance was examined in GraphPad Prism 7 (GraphPad Software, Inc., La Jolla, CA, USA) through one-way analysis of variance (ANOVA) and Duncan’s test at *p* ≤ 0.05. Correlation and clustering analysis was conducted by R version 3.5.3.

## 3. Results

### 3.1. Proximate Composition Analysis of PSK and PSC

Seed proximate composition of PSK and PSC from 11 production areas is shown in Table 1, which indicates that significant differences existed in detected samples. Seed neatness ranged from 93.08 ± 1.30% (P6) to 99.84 ± 0.12 % (P11), with an average of 98.11 ± 1.85%, and 100-seed weight varied from 16.08 ± 0.51 g (P10) to 32.88 ± 0.15 g (P11), with an average of 24.02 ± 4.64 g. As *P. ostti* seed consisted of two parts, seed kernel and coat, the seed coat rate was calculated. The results indicated that the average seed coat rate was 35.17 ± 2.64% and ranged from 31.13 ± 0.60% (P2) to 38.95 ± 0.07% (P10), which indicated that the coat accounted for 1/3 of the seed. Meanwhile, we found that the average moisture rate of PSC (7.35 ± 1.00%) was higher than that of PSK (4.04 ± 0.51%). The average crude protein (20.21 ± 1.65%) in PSK was much higher than that in PSC (3.70 ± 0.55%), which suggested that PSK could also be a good source of protein supply for further food processing. Seeds of P10 showed the highest content of crude protein both in PSC (4.62 ± 0.03%) and PSK (22.73 ± 0.15%) compared with those of other areas, while P7 (3.05 ± 0.06%) and P6 (16.91 ± 0.15%) showed the lowest ones in PSC and PSK.

### 3.2. Composition of the Fatty Acids in the PSK and PSC 

**Figure 2:**
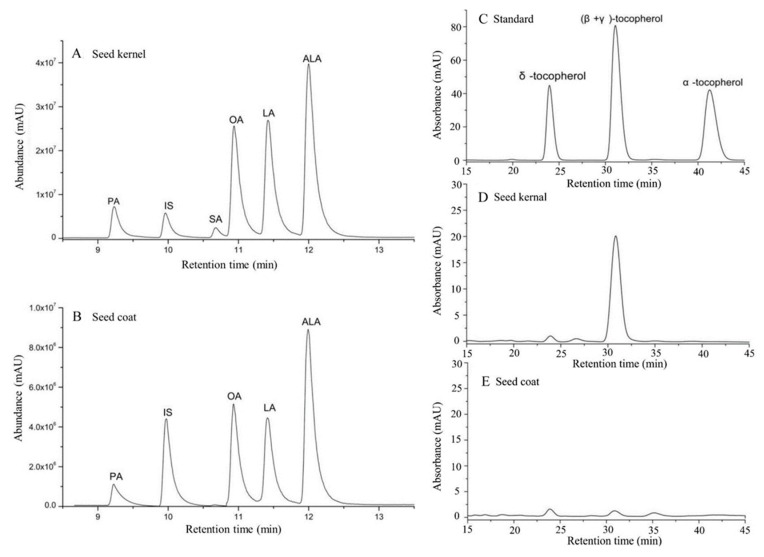
Chromatograms of fatty acid methyl esters (FAMEs) and tocopherol component from *P. ostii* seeds. (**A**,**B**): FAMEs; (**C**–**E**): tocopherol standards, tocopherol in seed coat and seed kernel, respectively. Peaks: PA = C16: 0 (Palmitic Acid); IS = C17: 0 (Internal Standard); SA = C18: 0 (Stearic Acid); OA = C18: 1∆9c (Oleic Acid); LA = C18: 2∆9c, 12c (Linoleic Acid); ALA = C18: 3∆9c, 12c, 15c (α-Linolenic Acid).

**Figure 3:**
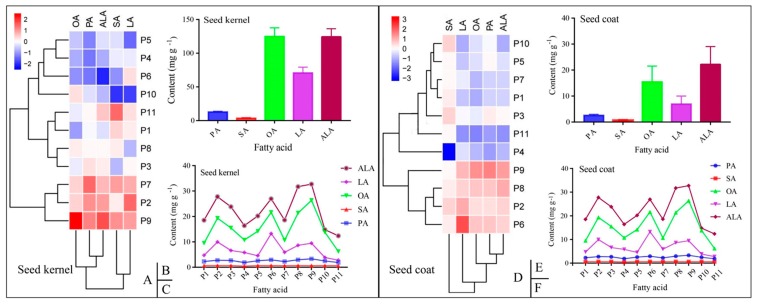
The heatmap and variation tendency of fatty acids (FAs) in seed kernel and seed coat from 11 production areas. (**A**,**D**): the heatmap of fatty acids in seed kernel and seed coat, respectively; (**B**,**E**): the content of five main fatty acids in seed kernel and seed coat, respectively; (**C**,**F**): variation tendency of fatty acids in seed kernel and seed coat from 11 production areas, respectively. PA = C16: 0 (Palmitic Acid); SA = C18: 0 (Stearic Acid); OA = C18: 1∆9c (Oleic Acid); LA = C18: 2∆9c, 12c (Linoleic Acid); ALA = C18: 3∆9c, 12c, 15c (α-Linolenic Acid).

The composition of FAs in PSC and PSK from 11 production areas were analyzed by GC-MS. In total, five fatty acids including palmitic (C16: 0; PA), stearic (C18: 0; SA), oleic (C18: 1; OA), linoleic (C18: 2; LA), and α-linolenic (C18: 3; ALA) acids were identified by comparison to retention time of FA standards (Figure 2A,B). A heat map based on the content of the five FAs was constructed (Figure 3A,D), and in PSK, the 11 detected samples were clustered into three groups, namely, samples from P2, P7, and P9 were clustered together due to their high content of OA, LA, and ALA, while a similar trend was observed in P2, P6, P8, and P9 in PSC. OA and ALA were both dominant FAs in PSK and PSC (Figure 3B,E), and significant differences in the content of the five FAs among the 11 production areas existed in PSC or PSK (Figure 3C,F).

### 3.3. Content of the Five Mainfas in PSK and PSC

The average total fatty acid (TFA) content was 335.79 ± 43.59 (298.89–399.34) mg g^−1^ in PSK, which was much higher than that of PSC (47.62 ± 18.42 mg g^−1^), while the average unsaturated fatty acids (UFA) content was 319.60 ± 41.74 mg g^−1^ and 44.46 ± 17.84 mg g^−1^ in PSK and PSC, respectively (Table 2), which accounted for 95.16% of TFAs in PSK, and was a little bit higher than that found in PSC (92.78%) (Table S2). Furthermore, the polyunsaturated fatty acids (PUFAs) ranged from 21.35 ± 4.22 mg g^−1^ (P11) to 68.61 ± 14.66 mg g^−1^ (P9) in PSC and from 296.56 ± 38.59 mg g^−1^ (P5) to 381.27 ± 69.99 mg g^−1^ (P9) in PSK (Table 2).

In PSK, among the saturated fatty acids (SFAs), PA content was higher than that of SA, which accounted for 3.79 ± 0.17% and 1.03 ± 0.15% of TFA, respectively (Table S2). While, among UFAs; OA, ALA, and LA accounted for 37.12 ± 1.88%, 37.01 ± 1.49%, and 21.03 ± 2.06% of TFAs, respectively (Table 2 and Table S2). The ratio of n-6:n-3 was between 0.47–0.74, with an average of 0.57 from 11 production areas; P10 possessed the lowest ratio, while P6 had the highest one (Table 2). Similarly to PSK, PA content was higher than that of SA in PSC, which accounted for 5.72 ± 1.22% and 1.49 ± 0.56% of TFA, respectively (Table S2). Among the three main UFAs, the highest content of OA, LA, and ALA was obtained in P9 (26.40 ± 5.44 mg g^−1^), P6 (13.26 ± 3.27 mg g^−1^), and P9 (32.73 ± 6.45 mg g^−1^), respectively (Table 2). Among the 11 production areas, the ratio of n-6:n-3 was between 0.22–0.49, with an average of 0.31 from the 11 production areas, among which P11 possessed the lowest ratio, while P6 had the highest one (Table 2). From the results, it can be observed that P9 had the greatest amount of UFAs (OA and ALA) both in PSK and PSC. 

**Figure 4:**
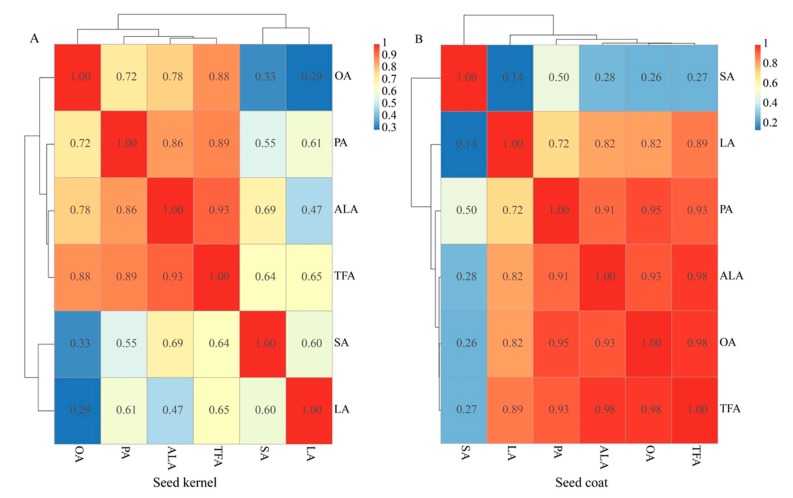
Correlation analysis on the content of total fatty acids (TFAs) and each kind of fatty acid including PA, SA, OA, LA, and ALA in seed coat and seed kernel. (**A**): Seed coat; (**B**): Seed kernel. PA = C16: 0 (Palmitic Acid); SA = C18: 0 (Stearic Acid); OA = C18: 1∆9c (Oleic Acid); LA = C18: 2∆9c, 12c (Linoleic Acid); ALA = C18: 3∆9c, 12c, 15c (α-Linolenic Acid).

The correlation analysis of the five obtained FAs and TFAs was conducted using R version 3.5.3, and indicated that TFA significantly related to each kind of FA (PA, OA, LA, ALA. and SA) in PSK (Figure 4A) and PSC (except SA) (Figure 4B). The correlation of ALA and TFA was 0.93 and higher than that of others in PSK, followed by PA, which was highly correlated with ALA; while in PSC, OA and ALA had higher correlation with TFA and reached about 0.98, and PA and OA demonstrated higher correlation against other kind of FAs, since seed coat was the maternal origin. The difference between PSK and PSC may be due to their genomic background.

### 3.4. Compounds and Content Analysis of Phytochemical in PSK and PSC

**Figure 5:**
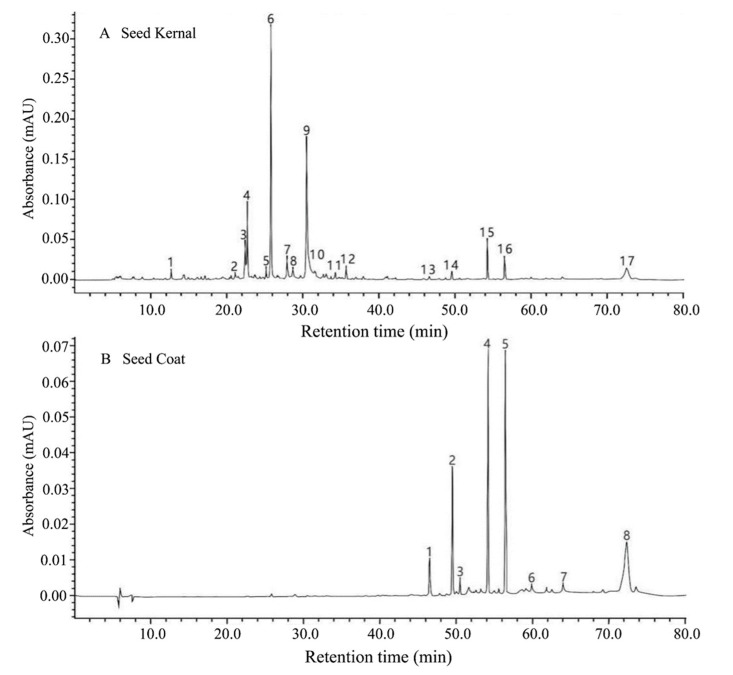
HPLC chromatograms of chemicals in the kernel and coat of the *P. ostii* seed from 11 production areas at 280 nm.

In the present study, a total of 17 compounds were detected from the seed kernel and seed coat by HPLC (at 280 nm) (Figure 5), among which nine were putatively identified and included: Oxypaeoniflora, 6′-O-β-Glucopyranosylalbiflorin, β-Gentiobiosylpaeoniflorin, Albiflorin, Paeoniflorin, Suffruticosol A, Suffruticosol B, trans-ε-Viniferin, and Suffruticosol C (Table 3). Based on the semi-quantitative method, the content of these compounds was analyzed, and it was observed that the PSK contained more components with an extremely lower content ranging from 0.44 ± 0.34 mg g^−1^ (Suffruticosol A) to 17.87 ± 5.71 mg g^−1^ (Paeoniflorin). While in PSC, a higher content of peak 8-c was found, with an average of 362.89 ± 35.05 mg g^−1^, followed by Suffruticosol C (341.16 ± 43.66 mg g^−1^, on average), and trans-ε-Viniferin (202.97 ± 82.61 mg g^−1^, on average). There were significant differences among samples from the 11 production areas (Table 4 and Table S3).

### 3.5. Antioxidant Activity Analysis of PSK and PSC 

**Figure 6:**
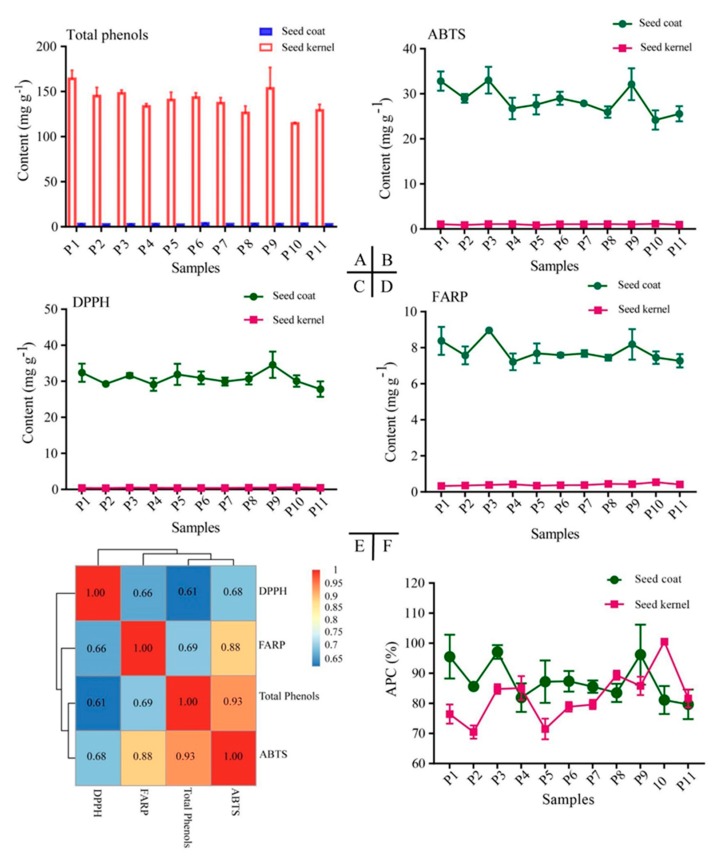
Total phenol content and antioxidant activity of seed kernel and coat of *P. ostii* from 11 production areas. (**A**): Total phenol content; (**B**–**D**): = 2,2′-azinobis-(3-ethylbenzothiazoline-6-sulfonic acid) assay (ABTS), 1,1-diphenyl-2-picrylhydrazyl (DPPH) and ferric reducing antioxidant power (FRAP) assays; (**E**): Correlation analysis on total phenol content and values of ABTS, DPPH, and FRAP; (**F**): Antioxidant potency composite (APC) analysis.

Oxidative stability provides a good estimation for the susceptibility of oils and fats to oxidation. Total phenols were analyzed, and a high content of total phenols (an average of 139.49 ± 15.40 mg g^−1^) was found in PSC, ranging from 114.57 ± 1.11 mg g^−1^ (P10) to 164.08 ± 9.58 mg g^−1^ (P1). Significantly lower phenolic content was found in PSK (an average of 3.04 ± 0.41 mg g^−1^), ranging from 2.35 ± 0.27 mg g^−1^ (P7) to 3.70 ± 0.61 mg g^−1^ (P8). Significant differences in total phenol content were observed among PSC from the detected samples, while PSK demonstrated no obvious differences (Table 5, Figure 6A).

Furthermore, DPPH, FRAP, and ABTS assays were conducted to assess the oxidative stability of PSC and PSK in all samples. The average antioxidant activity detected using DPPH, FRAP, and ABTS assays was 30.75 ± 2.47, 7.76 ± 0.64, and 28.53 ± 3.40 mg g^−1^ in PSC and 0.43 ± 0.01, 0.4 ± 0.06, and 1.02 ± 0.10 mg g^−1^ in PSK, respectively, which suggested that PSC exhibited significantly higher oxidative stability than PSK (Table 5, Figure 6B–D). Samples of P10 demonstrated the highest antioxidant activity in PSK. In PSC, the highest values were observed in P9 (34.57 ± 3.63 mg g^−1^, DPPH) and P3 (8.95 ± 0.18, 33.00 ± 2.97 mg g^−1^, FRAP and ABTS) (Table 5; Figure 6B–D). Although there was a little bit of discrepancy among various regions, a similar trend and positive strong correlation was found between the DPPH, FRAP, and ABTS assays and the total phenol content in PSC (Figure 6E). In order to comprehensively compare the antioxidant activity between PSK and PSC from 11 production areas, the APC index was calculated (Table S4), and the ranked antioxidant activity from the largest to the smallest was as follows: P3 > P9 > P1 > P6 > P5 > P2 > P7 > P8 > P4 > P10 > P11 (PSC) and P10 > P8 > P9 > P4 > P3 > P11 > P7 > P6 > P1 > P5 > P2 (PSK) (Figure 6F, Table S4).

### 3.6. Tocopherols Content Analysis in PSK and PSC

There was a total of 3 tocopherols isomers detected by HPLC in PSK (β-, γ-, and δ- tocopherol), while no isomers were detected in PSC from all samples (Figure 2C–E). Tocopherols (β- + γ-) were the predominant tocopherol homologs (216.37 ± 37.05 μg g^−1^, average) (Table 6), and trace δ-tocopherols (6.05 ± 1.23 μg g^−1^) was detected in PSK. The highest total tocopherols (276.70 ± 2.5 μg g^−1^) was observed in P1 samples, whereas, P6 showed the lowest content (167.83 ± 5.83 μg g^−1^). Significant differences were obtained among detected samples from the 11 production areas (Table 6).

## 4. Discussion

The present study focused on the characterization of nutritional data, including fatty acids, crude proteins, tocopherol, phytochemical compounds, and antioxidants of PSK and PSC from 11 production areas. The data from this study will provide a direction for the selection of high-quality seeds for oil production and a strategy for making full use of seeds in the food industry. 

Phenotypic related traits have a certain relationship with seed quality. Seed neatness, moisture content, 100-seed weight, and seed coat rate were characterized in the present study. Of these traits, 100-seed weight is an index that reflects the size and fullness of the seeds and has been shown to be a predictor of production [29]. In the present study, the 100-seed weight ranged from 16.08 g to 32.89 g among sampled seeds from 11 production areas, which suggested variation did exist, and would provide a basis for target region selection. Seed coat rate is directly related to oil production, and high seed coat rate (35.17%) accounted for about 1/3 of the total seed mass obtained in the present study, which provides a potential use for remnants after oil processing. In future studies, the breeding of new cultivars with lower seed coat rate would be a promising prospect in view of edible oil production. Similar results of seed coat rate have been obtained in *Lupinus angustifolius* [30], rapeseed [31], and beans [32], which would function as good references for tree peony breeding with the intent of producing higher oil content. In addition, the relatively high level of crude protein in seed kernel (20.21%) indicates that the seed could be included in food formulations as a source of protein after oil processing. 

FAs consisted of SFAs and UFAs, and the latter are divided into monounsaturated (MUFAs) and PUFAs, which are the most important nutritional components of edible oil or other functional foods [33]. PUFAs are essential fatty acids (EFAs) and present as the predominant part of FAs in *P. ostii* seeds, among which ALA was significantly abundant. In contrast, compared with other common edible oils, ALA was less than 10%, such as in soybean oil (8.95%), canola oil (9.74%), corn germ oil (0.55%), sesame oil (0.32%), and olive oil (0.57%) [11], which indicated that *P. ostii* seed oil is a good source for ALA supplementation. ALA is also a precursor for docosahexaenoic acid (DHA) and eicosapentaenoic acid (EPA), which have been demonstrated as protection agents against chronic daily headaches, aging and dementia, and metal disorders [34,35,36]. 

In addition, seed oil of *P. ostii* is unique for its n-6:n-3 FA ratio (LA/ALA), which is lower than 1.00. Since n-6 and n-3 PUFAs demonstrated significant differences in biological function, the n-6:n-3 FA ratio has been suggested to be a key factor for the balance of dietary patterns and synthesis of eicosanoids, which is of significant nutritional importance [35,37]. Due to traditional dietary habits, the ratio of n-6:n-3 FA reached 15–20:1 as a result of a decreased supply of n-3 FAs, which have been related to a series of chronic diseases, such as atherosclerosis, essential hypertension, obesity, diabetes, arthritis, and other autoimmune diseases, as well as cancers of the breast, colon, and prostate [10,34]. Therefore, a lower n-6:n-3 FA ratio is preferred for modern diets when considering nutritional and health benefits [34]. Thus, edible oil like *P. ostii* seed oil with a higher content of ALA and a lower ratio of n-6:n-3 FA would be more desirable for diets to reduce the risk of many chronic diseases.

Tocopherols have commanded the most interest because of their availability and overall health impact, and their central role in preventing oxidation at the cellular level [38]. Tocopherols are a group of fat-soluble antioxidants and can be divided into α-, β-, γ-, and δ- forms [39]. In present study, the α-tocopherols were not detected in tested *P. ostii* seed. Similarly to previous studies, γ-tocopherols were the main type in PSK, with significantly higher levels than that of other tocopherol components [40,41]. The largest extracted amount of the total tocopherols in *P. ostii* seed was about 276.7 μg g^−1^ DW (P1) in this study, which is lower than that in soybean (664 μg g^−1^ DW) [42], rapeseed (460.07 μg g^−1^ DW) [43], and sunflower seed (555.2 μg g^−1^ DW) [44], but higher than that in *Triticum* species, such as *T. thaoudar*, *T. aegilopoides*, *T. monococcum*, and *T. urartu* (75.1, 70.8, 66.8 and 63.9 μg g^−1^ DW, respectively) [45,46]. Although the content of tocopherols is moderate, *P. ostii* seed would be a good source of tocopherol supply.

Oxidative stability of vegetable oils is significantly impacted by the FA composition, and the oxidation rates of individual FAs have been confirmed to be related to the degree of unsaturation. For OA (C18: 1), LA (18: 2), and ALA (C18: 3) in normal autoxidation reactions, the ratio of oxidation rates has been shown to be 1:12:25 [47]. Tocopherols are essential for the protection of PUFAs against peroxidation in plants and animals due to their action in scavenging active oxygen species and free radicals. They also perform as efficient terminators in the lipid autoxidation reaction process [48]. Vegetable oils are notable as major sources of dietary PUFAs and tocopherols. In sunflower oil, OA and LA content accounted for 90.2% of total FAs [49], and three kinds (α, β, and γ) of tocopherols were detected, and α-tocopherols (671 ppm) were abundant. In contrast, in soybean oil, the total content of OA and LA was 76% of total FAs, and four kinds of tocopherols were obtained [50] (β + γ- (595 ppm), and δ- tocopherols (263 ppm) were abundant. In olive seed oil, OA and LA were rich and accounted for 80.6% [51], and α, β and γ-tocopherols were detected (α-(96 ppm) was abundant and β + γ- was 18 ppm). In perilla seed oil, ALA was 62.6%, and the abundant tocopherols were the γ- ones (526 ppm, β + γ-) [52]. It has been demonstrated that a positive correlation between ALA and γ-tocopherol existed, and oils rich in ALA had low α-tocopherol content [53]. Similarly, in this study, OA, ALA, and LA accounted for 95.16% of total FAs in *P. ostii* seed, and α-tocopherol was almost undetected, while β + γ tocopherol content ranged from 16.08 to 26.81 mg g^−1^. This would be a good protector for *P. ostii* seed oil from the oxidative deterioration of the essential components of nutrients, since fat-soluble vitamins could prevent or delay lipid oxidation in seed oils [47].

The presence of natural antioxidants in plants is well known and has various uses. The antioxidant effects of several substances in plants, such as polyphenols, have been reported to have multiple biological effects [53]. In the present study, total phenols were significantly higher in seed coats and had a positive correlation with DPPH (*r* = 0.61), FRAP (*r* = 0.69), and ABTS (*r* = 0.93) (Figure 6E), which suggested that they contributed significantly to the antioxidant activity. The phenolic compounds are increasingly of interest in the food industry because they retard the oxidative degradation of lipids and thereby improve the quality and nutritional value of food [54]. High amounts of secondary metabolites were also detected in seed coat, and we putatively identified nine chemicals. Similar results were also obtained in root bark in our former study [55], which demonstrated high activities as an analgesic, sedative, and anti-inflammatory agent and performed as a good remedy for cardiovascular disease, stagnated blood, and brain injury in traditional oriental medicine [56,57,58]. Therefore, the seed coat of *P. ostii* could be a potential source of natural antioxidants in food and pharmaceutical applications.

High quality seeds are the most basic and important element of agricultural and industrial production. *P. ostii* is a new emerging woody oil crop, and the seed quality is a decisive factor for the development of the industry. In the present study, seeds from P9 showed the greatest amount of OA, LA, ALA, UFAs, PUFAs, and TFAs, followed by P2, both in PSC and PSK, which indicated that the P9 would be the most promising high-quality production area for the industrial purposes of the *P. ostii* seed. According to the above results of this study, the use of *P. ostii* seeds should be encouraged, since they present a high content of crude protein, ALA, PUFAs, and tocopherols, which are beneficial to human health. This study can guide producers to make a strategy for the full use of *P. ostii* seeds, not only for edible oil, but also for functional food.

## 5. Conclusions

In this study, a comprehensive analysis on seed quality traits was conducted on *P. ostii* from 11 various production areas, which demonstrated significant differences in basic proximate composition and nutrient components, especially between PSC and PSK. The FA composition of PSC and PSK was the same, which included PA, SA, OA, LA, and ALA, while the contents of TFAs or single kind of FAs varied significantly and were higher in PSK as compared with PSC. The tocopherol was mainly detected in PSK, and (β + γ)-tocopherol was dominant. A total of 17 compounds were detected in PSC and PSK, and the content of these compounds was much higher in PSC, and significant differences existed among samples. Obvious differences in antioxidant activity among samples were detected by DPPH, ABTS, and FRAP assays, and PSC demonstrated much higher antioxidant potential than that of PSK. P9 would be the most promising production area for industrial purposes. This study provides a basis for seed quality control and making full use of seeds as good source of valuable oil and functional food.

## Figures and Tables

**Table 1:** The proximate composition and content of the *P. ostii* seeds from 11 production areas.

Code	Neatness (%)	100-Seed Weight (g)	Seed Coat Rate (%)	Moisture Content (%)		Crude Protein Content (%)	
				Seed Coat	Seed Kernel	Seed Coat	Seed Kernel
P1	98.67 ± 0.23 ^d^	30.25 ± 0.48 ^h^	34.15 ± 0.05 ^bc^	7.41 ± 0.28 ^bcd^	4.25 ± 0.02 ^cd^	3.47 ± 0.08 ^c^	18.65 ± 0.13 ^b^
P2	97.29 ± 0.30 ^b^	28.15 ± 0.34 ^g^	31.13 ± 0.60 ^a^	6.40 ± 0.28 ^ab^	3.57 ± 0.05 ^a^	4.09 ± 0.04 ^f^	21.33 ± 0.14 ^e^
P3	99.62 ± 0.08 ^f^	22.26 ± 0.48 ^d^	31.62 ± 0.79 ^a^	6.14 ± 0.39 ^a^	3.49 ± 0.14 ^a^	3.60 ± 0.08 ^cd^	19.78 ± 0.21 ^c^
P4	97.64 ± 0.21 ^bc^	24.02 ± 0.41 ^f^	35.52 ± 1.78 ^cd^	8.69 ± 0.15 ^e^	4.36 ± 0.03 ^cd^	3.68 ± 0.04 ^d^	20.15 ± 0.11 ^d^
P5	99.59 ± 0.07 ^f^	22.41 ± 0.26 ^d^	32.82 ± 0.39 ^ab^	7.73 ± 0.60 ^cde^	5.00 ± 0.11 ^e^	3.80 ± 0.03 ^e^	21.39 ± 0.18 ^e^
P6	93.08 ± 1.30 ^a^	21.71 ± 0.48 ^c^	36.38 ± 1.75 ^de^	7.92 ± 0.48 ^cde^	3.91 ± 0.29 ^abc^	3.51 ± 0.02 ^c^	16.91 ± 0.15 ^a^
P7	98.80 ± 0.24 ^d^	23.15 ± 0.34 ^e^	35.15 ± 0.53 ^cd^	7.44 ± 0.77 ^bcd^	4.07 ± 0.10 ^bc^	3.05 ± 0.06 ^a^	20.04 ± 0.14 ^cd^
P8	98.70 ± 0.21 ^d^	24.13 ± 0.32 ^f^	35.26 ± 1.06 ^cd^	8.63 ± 1.77 ^de^	3.53 ± 0.21 ^a^	3.66 ± 0.04 ^d^	18.82 ± 0.08 ^b^
P9	98.37 ± 0.08 ^cd^	19.22 ± 0.56 ^b^	37.24 ± 0.70 ^e^	6.43 ± 0.23 ^ab^	3.72 ± 0.32 ^ab^	4.46 ± 0.09 ^g^	20.23 ± 0.27 ^d^
P10	97.59 ± 0.42 ^bc^	16.08 ± 0.51 ^a^	38.95 ± 0.07 ^f^	7.17 ± 0.15 ^abc^	4.60 ± 0.11 ^de^	4.62 ± 0.03 ^h^	22.73 ± 0.15 ^f^
P11	99.84 ± 0.12 ^f^	32.88 ± 0.15 ^i^	38.64 ± 0.81 ^ef^	6.86 ± 0.25 ^abc^	3.95 ± 0.66 ^abc^	3.20 ± 0.17 ^b^	22.27 ± 0.14 ^g^
Range	93.08–99.84	16.08–32.88	31.13–38.95	6.14–8.69	3.49–5.00	3.05–4.62	16.91–22.73
Average	98.11 ± 1.85	24.02 ± 4.64	35.17 ± 2.64	7.35 ± 0.10	4.04 ± 0.51	3.70 ± 0.55	20.21 ± 1.65

Note: Data were represented as mean of three different determinations ± standard deviation. Different tiny letters in the same row indicate significant differences of *p* ≤ 0.05.

**Table 2:** Fatty acid composition and content in kernel and coat of *P. ostii* seeds from 11 production areas.

		P1	P2	P3	P4	P5	P6	P7	P8	P9	P10	P11
PA	PSC	2.27 ± 0.26 ^ab^	2.75 ± 0.15 ^abc^	2.69 ± 0.24 ^abc^	1.86 ± 1.41 ^a^	2.55 ± 0.15 ^abc^	2.90 ± 0.42 ^bc^	2.25 ± 0.39 ^ab^	2.94 ± 0.10 ^bc^	3.32 ± 0.43 ^c^	2.47 ± 0.13 ^abc^	1.88 ± 0.20 ^a^
	PSK	12.71 ± 0.67 ^abc^	13.84 ± 0.54 ^abc^	13.11 ± 0.67 ^abc^	11.31 ± 1.19 ^a^	11.43 ± 0.36 ^ab^	11.32 ± 0.72 ^a^	14.27 ± 1.87 ^c^	12.82 ± 1.78 ^abc^	14.04 ± 2.53 ^bc^	12.32 ± 0.64 ^abc^	12.76 ± 1.85 ^abc^
SA	PSC	0.63 ± 0.02 ^a^	0.65 ± 0.01 ^a^	0.64 ± 0.02 ^a^	0.46 ± 0.34 ^a^	0.63 ± 0.03 ^a^	0.64 ± 0.02 ^a^	0.63 ± 0.02 ^a^	0.64 ± 0.01 ^a^	0.62 ± 0.01 ^a^	0.65 ± 0.01 ^a^	0.63 ± 0.00 ^a^
	PSK	3.72 ± 0.31 ^bcd^	3.53 ± 1.02 ^bcd^	3.07 ± 0.56 ^abc^	3.34 ± 0.34 ^abcd^	3.32 ± 0.22 ^abcd^	2.85 ± 0.57 ^ab^	4.00 ± 0.36 ^cd^	3.57 ± 0.60 ^bcd^	4.02 ± 0.65 ^cd^	2.42 ± 0.08 ^a^	4.24 ± 0.50 ^d^
OA	PSC	9.51 ± 1.14 ^ab^	19.34 ± 3.76 ^cde^	15.51 ± 1.49 ^bcd^	10.76 ± 8.35 ^ab^	14.21 ± 0.79 ^bcd^	21.63 ± 5.31 ^de^	10.64 ± 4.05 ^ab^	21.41 ± 3.34 ^de^	26.40 ± 5.44 ^e^	13.81 ± 1.39 ^bc^	6.20 ± 1.59 ^a^
	PSK	112.56 ± 5.23 ^a^	129.67 ± 10.57 ^a^	122.85 ± 10.97 ^a^	113.24 ± 15.25 ^a^	116.46 ± 8.78 ^a^	111.25 ± 10.66 ^a^	129.75 ± 18.07 ^a^	126.63 ± 19.93 ^a^	157.63 ± 27.49 ^b^	129.29 ± 6.04 ^a^	121.97 ± 16.90 ^a^
LA	PSC	4.71 ± 0.29 ^ab^	9.98 ± 1.54 ^cd^	6.60 ± 1.18 ^abc^	5.80 ± 4.57 ^abc^	4.54 ± 1.28 ^ab^	13.26 ± 3.27 ^d^	5.90 ± 2.52 ^abc^	8.64 ± 1.30 ^bc^	9.48 ± 2.77 ^cd^	3.86 ± 0.66 ^a^	2.76 ± 0.59 ^a^
	PSK	72.41 ± 3.98 ^bcd^	83.59 ± 7.05 ^d^	70.95 ± 5.96 ^abcd^	69.05 ± 10.49 ^abcd^	58.20 ± 7.00 ^ab^	73.61 ± 6.29 ^bcd^	77.41 ± 11.81 ^cd^	64.50 ± 11.02 ^abc^	78.92 ± 15.10 ^cd^	54.39 ± 0.71 ^a^	73.88 ± 10.61 ^bcd^
ALA	PSC	18.55 ± 2.33 ^abc^	27.77 ± 3.93 ^cd^	23.85 ± 4.42 ^bcd^	16.40 ± 12.76 ^ab^	20.20 ± 3.01 ^abc^	26.98 ± 5.11 ^cd^	18.61 ± 5.37 ^abc^	31.72 ± 3.89 ^d^	32.73 ± 6.45 ^d^	14.80 ± 1.53 ^ab^	12.38 ± 2.06 ^a^
	PSK	121.15 ± 4.12 ^abc^	135.06 ± 6.46 ^bc^	126.67 ± 5.13 ^abc^	114.27 ± 12.93 ^ab^	120.52 ± 10.17 ^abc^	99.85 ± 8.71 ^a^	132.69 ± 18.07 ^bc^	124.77 ± 18.88 ^abc^	144.71 ± 27.45 ^c^	116.60 ± 2.66 ^abc^	130.98 ± 20.75 ^bc^
TFA	PSC	35.69 ± 3.94 ^ab^	60.50 ± 9.37 ^cde^	49.32 ± 6.50 ^bcd^	35.30 ± 27.41 ^ab^	42.14 ± 4.92 ^abc^	65.43 ± 14.08 ^de^	38.05 ± 12.30 ^ab^	65.37 ± 8.60 ^de^	72.56 ± 15.10 ^e^	35.60 ± 3.67 ^ab^	23.87 ± 4.42 ^a^
	PSK	322.58 ± 11.56 ^a^	365.72 ± 23.34 ^ab^	336.67 ± 23.03 ^ab^	311.23 ± 40.09 ^a^	309.94 ± 26.33 ^a^	298.89 ± 26.64 ^a^	358.13 ± 49.87 ^ab^	332.32 ± 51.55 ^ab^	399.34 ± 73.16 ^b^	315.05 ± 9.37 ^a^	343.85 ± 49.73 ^ab^
UFA	PSC	32.78 ± 3.69 ^ab^	57.10 ± 9.23 ^cde^	45.97 ± 6.64 ^bcd^	32.97 ± 25.67 ^ab^	38.95 ± 5.05 ^abc^	61.89 ± 13.68 ^de^	35.17 ± 11.94 ^ab^	61.79 ± 8.50 ^de^	68.61 ± 14.66 ^e^	32.48 ± 3.58 ^ab^	21.35 ± 4.22 ^a^
	PSK	306.13 ± 11.35 ^a^	348.33 ± 21.89 ^ab^	320.48 ± 21.83 ^ab^	296.56 ± 38.59 ^a^	295.19 ± 25.93 ^a^	284.71 ± 25.59 ^a^	339.85 ± 47.91 ^ab^	315.92 ± 49.42 ^ab^	381.27 ± 69.99 ^b^	300.30 ± 8.80 ^a^	326.84 ± 47.43 ^ab^
PUFA	PSC	23.26 ± 2.61 ^ab^	37.76 ± 5.47 ^cd^	30.46 ± 5.59 ^bcd^	22.21 ± 17.34 ^ab^	24.74 ± 4.29 ^abc^	40.25 ± 8.38 ^d^	24.52 ± 7.89 ^abc^	40.37 ± 5.16 ^d^	42.21 ± 9.23 ^d^	18.66 ± 2.19 ^ab^	15.15 ± 2.64 ^a^
	PSK	193.57 ± 6.60 ^abc^	218.65 ± 13.15 ^bc^	197.62 ± 10.90 ^abc^	183.32 ± 23.34 ^abc^	178.72 ± 17.15 ^abc^	173.46 ± 14.99 ^ab^	210.10 ± 29.88 ^abc^	189.28 ± 29.86 ^abc^	223.64 ± 42.54 ^c^	171.00 ± 2.78 ^a^	204.86 ± 31.29 ^abc^
n-6/n-3	PSC	0.25	0.36	0.28	0.35	0.22	0.49	0.32	0.27	0.29	0.26	0.22
	PSK	0.60	0.62	0.56	0.60	0.48	0.74	0.58	0.52	0.55	0.47	0.56

Note: PSK = *P. ostii* Seed Kernel; PSC = *P. ostii* Seed Coat; PA = Palmitic Acid; SA = Stearic Acid; OA = Oleic Acid; LA = Linoleic Acid; ALA = α-Linolenic Acid; TFA = Total Fatty Acid; UFA = Unsaturated Fatty Acid; PUFA = Polyunsaturated Fatty Acid; n-6/n-3 = Linoleic Acid/α-Linolenic Acid. Data are represented as mean of three different determinations ± standard deviation. Different tiny letters in the same row indicate significant differences of *p* ≤ 0.05.

**Table 3:** The characterization and identification of compounds detected in *P. ostii* seeds.

No.	Peak	Retention Time (min)	λmax (nm)	Molecular Formula	[M + H]^+^ (*m/z*)	[M−H]^−^ (*m/z*)	[M + HCOO]^−^ (*m/z*)	[M + Na]^+^ (*m/z*)	Identification	Reference
1	3-k	22.4	258	C_23_H_27_O_12_	497	495		519	Oxypaeoniflora	[26]
2	6-k	25.8	238, 320	C_29_H_37_O_16_	643	641	687	665	6′-*O*-β-Glucopyranosylalbiflorin	[26]
3	7-k	27.9	238, 275	C_29_H_37_O_16_	643	687	611	665	β-Gentiobiosylpaeoniflorin	[26]
4	8-k	27.8	238, 275	C_23_H_27_O_11_	481	479	525	503	Albiflorin	[26]
5	9-k	30.5	238, 275	C_23_H_27_O_11_	481	479	525	503	Paeoniflorin	[26]
6	13-k, 1-c	46.0	243, 281	C_42_H_33_O_9_	681	679	725		SuffruticosolA	[27]
7	14-k, 2-c	49.5	243, 281	C_42_H_33_O_9_	681	679	725		SuffruticosolB	[27]
8	15-k, 4-c	54.2	246, 325	C_28_H_22_O_6_	455	453		477	trans-*ε*-Viniferin	[28]
9	16-k, 5-c	56.4	245, 327	C_42_H_33_O_9_	681	679	725		Suffruticosol C	[27]

Note: c, k indicates seed coat and seed kernel.

**Table 4:** The compound in seed coat of *P. ostii* from 11 production areas.

	P1	P2	P3	P4	P5	P6	P7	P8	P9	P10	P11
Peak 1	72.28 ± 0.14 ^h^	36.19 ± 1.32 ^a^	46.02 ± 0.33 ^c^	46.70 ± 0.64 ^c^	44.05 ± 0.30 ^b^	61.33 ± 0.21 ^f^	55.61 ± 0.99 ^e^	50.18 ± 0.96 ^d^	75.14 ± 1.42 ^i^	43.02 ± 0.34 ^b^	70.58 ± 1.13 ^g^
Peak 2	222.25 ± 0.35 ^i^	105.46 ± 0.37 ^a^	140.61 ± 1.29 ^d^	134.02 ± 0.80 ^c^	121.95 ± 0.22 ^b^	185.26 ± 1.52 ^f^	160.10 ± 1.17 ^e^	159.55 ± 1.13 ^e^	197.28 ± 2.29 ^g^	106.74 ± 0.74 ^a^	204.05 ± 1.24 ^h^
Peak 3	21.78 ± 0.35 ^f^	11.24 ± 0.04 ^a^	14.09 ± 0.24 ^cd^	13.83 ± 0.07 ^cd^	13.39 ± 0.08 ^bc^	17.04 ± 0.40 ^e^	16.57 ± 0.43 ^e^	14.67 ± 0.84 ^d^	22.69 ± 1.18 ^g^	12.73 ± 0.29 ^b^	21.50 ± 0.57 ^f^
Peak 4	81.86 ± 1.78 ^a^	164.83 ± 0.55 ^d^	254.93 ± 1.37 ^h^	159.80 ± 0.51 ^c^	171.93 ± 1.50 ^e^	151.96 ± 1.08 ^b^	212.17 ± 0.93 ^g^	174.17 ± 0.98 ^f^	318.39 ± 1.66 ^i^	383.00 ± 1.40 ^j^	159.57 ± 1.26 ^c^
Peak 5	326.79 ± 4.88 ^d^	344.19 ± 0.67 ^e^	346.04 ± 0.78 ^e^	352.59 ± 2.05 ^f^	240.44 ± 0.56 ^a^	312.34 ± 1.46 ^c^	383.56 ± 1.59 ^h^	377.75 ± 0.93 ^g^	379.11 ± 1.05 ^g^	298.32 ± 0.70 ^b^	391.66 ± 0.52 ^i^
Peak 6	16.79 ± 0.71 ^b^	17.95 ± 0.45 ^b^	16.78 ± 0.35 ^b^	16.67 ± 0.41 ^b^	16.49 ± 0.12 ^b^	11.76 ± 0.31 ^a^	16.67 ± 0.62 ^b^	17.65 ± 0.87 ^b^	16.98 ± 0.56 ^b^	21.49 ± 2.05 ^c^	20.79 ± 1.39 ^c^
Peak 7	62.06 ± 0.87 ^e^	24.98 ± 0.64 ^a^	57.52 ± 4.00 ^d^	24.97 ± 0.11 ^a^	25.55 ± 0.54 ^a^	31.60 ± 1.08 ^c^	24.33 ± 0.58 ^a^	25.00 ± 0.52 ^a^	23.82 ± 0.72 ^a^	28.54 ± 0.83 ^b^	25.22 ± 0.17 ^a^
Peak 8	332.11 ± 1.40 ^b^	331.53 ± 0.28 ^b^	328.87 ± 0.87 ^b^	368.61 ± 1.31 ^d^	368.50 ± 0.93 ^d^	369.62 ± 1.09 ^d^	317.94 ± 1.40 ^a^	357.46 ± 1.14 ^c^	386.72 ± 6.74 ^e^	385.49 ± 2.10 ^e^	444.96 ± 1.61 ^f^

Note: Data are represented as mean of three different determinations ± standard deviation. Different tiny letters in the same row indicate significant differences of *p* ≤ 0.05.

**Table 5:** Antioxidant activities of the seed kernel and coat of the *P. ostii* from 11 production areas.

Code		P1	P2	P3	P4	P5	P6	P7	P8	P9	P10	P11
TP	PSC	164.08 ± 9.58 ^f^	144.92 ± 9.66 ^cde^	147.85 ± 3.72 ^def^	133.35 ± 3.16 ^bcd^	140.63 ± 8.54 ^bcde^	143.32 ± 5.19 ^bcde^	137.07 ± 6.21 ^bcde^	126.23 ± 7.71 ^ab^	153.33 ± 23.43 ^ef^	114.57 ± 1.11 ^a^	129.01 ± 6.78 ^abc^
	PSK	3.10 ± 0.28 ^bc^	2.68 ± 0.10 ^ab^	2.91 ± 0.27 ^bc^	3.14 ± 0.24 ^bc^	2.35 ± 0.27 ^a^	3.70 ± 0.61 ^d^	3.02 ± 0.12 ^bc^	3.26 ± 0.17 ^cd^	3.04 ± 0.28 ^bc^	3.35 ± 0.11 ^cd^	2.93 ± 0.22 ^bc^
DPPH assay	PSC	32.38 ± 2.51 ^bc^	29.25 ± 0.78 ^ab^	31.59 ± 0.66 ^abc^	29.12 ± 1.76 ^ab^	31.91 ± 2.94 ^bc^	30.95 ± 1.76 ^abc^	29.92 ± 1.10 ^ab^	30.69 ± 1.61 ^abc^	34.57 ± 3.63 ^c^	30.07 ± 1.58 ^ab^	27.82 ± 2.15 ^a^
	PSK	0.40 ± 0.03 ^b^	0.36 ± 0.01 ^a^	0.46 ± 0.01 ^cd^	0.43 ± 0.05 ^bc^	0.40 ± 0.02 ^b^	0.40 ± 0.01 ^b^	0.41 ± 0.01 ^b^	0.48 ± 0.02 ^d^	0.46 ± 0.02 ^cd^	0.54 ± 0.01 ^e^	0.45 ± 0.01 ^cd^
FRAP assay	PSC	8.38 ± 0.78 ^bd^	7.57 ± 0.50 ^abc^	8.95 ± 0.18 ^d^	7.21 ± 0.46 ^a^	7.69 ± 0.55 ^abc^	7.59 ± 0.11 ^abc^	7.67 ± 0.18 ^abc^	7.44 ± 0.16 ^ab^	8.18 ± 0.84 ^bcd^	7.45 ± 0.35 ^ab^	7.27 ± 0.37 ^ab^
	PSK	0.32 ± 0.01 ^a^	0.35 ± 0.02 ^ab^	0.38 ± 0.02 ^bcde^	0.42 ± 0.02 ^def^	0.34 ± 0.02 ^ab^	0.36 ± 0.00 ^abc^	0.37 ± 0.01 ^abcd^	0.44 ± 0.05 ^f^	0.43 ± 0.05 ^ef^	0.53 ± 0.01 ^g^	0.40 ± 0.02 ^cdef^
ABTS assay	PSC	32.82 ± 2.13 ^cd^	28.98 ± 0.97 ^bc^	33.00 ± 2.97 ^d^	26.74 ± 2.38 ^ab^	27.58 ± 2.16 ^ab^	29.00 ± 1.46 ^bc^	27.89 ± 0.55 ^ab^	25.95 ± 1.27 ^ab^	32.11 ± 3.54 ^cd^	24.18 ± 2.13 ^a^	25.56 ± 1.67 ^ab^
	PSK	1.06 ± 0.09 ^de^	0.88 ± 0.04 ^ab^	1.07 ± 0.03 ^de^	1.08 ± 0.09 ^de^	0.84 ± 0.05 ^a^	1.05 ± 0.04 ^cde^	1.04 ± 0.02 ^cde^	1.06 ± 0.07 ^de^	1.02 ± 0.05 ^cd^	1.13 ± 0.00 ^e^	0.95 ± 0.03 ^bc^

Note: TP = total phenols. ABTS assay = 2,2′-azinobis-(3-ethylbenzothiazoline-6-sulfonic acid) assay, DPPH assay = 1,1-diphenyl-2-picrylhydrazyl assay; FRAP assay = ferric reducing antioxidant power (FRAP) assay. Data represent the mean of three different determinations ± standard deviation. Different letters in the same row indicate significant differences of *p* ≤ 0.05.

**Table 6:** The content of tocopherols in seed kernel of *P. ostii* from 11 production areas.

Code	(β- + γ-) Tocopherols (μg g^−1^)	δ-Tocopherol (μg g^−1^)	Total Tocopherols (μg g^−1^)
P1	268.16 ± 2.33 ^h^	8.54 ± 0.22 ^f^	276.70 ± 2.56 ^g^
P2	255.95 ± 1.95 ^g^	6.09 ± 0.09 ^cd^	262.04 ± 2.02 ^f^
P3	248.38 ± 7.94 ^g^	4.83 ± 0.22 ^a^	253.21 ± 8.17 ^f^
P4	230.21 ± 4.21 ^f^	5.65 ± 0.14 ^abc^	235.86 ± 4.31 ^e^
P5	245.40 ± 5.09 ^g^	5.02 ± 0.55 ^ab^	250.42 ± 5.17 ^f^
P6	160.88 ± 5.77 ^a^	6.95 ± 0.05 ^de^	167.83 ± 5.83 ^a^
P7	173.43 ± 7.83 ^c^	5.63 ± 0.12 ^abc^	179.06 ± 7.95 ^a^
P8	164.57 ± 14.25 ^ab^	6.02 ± 0.49 ^bcd^	170.59 ± 14.74 ^a^
P9	196.42 ± 2.97 ^d^	7.56 ± 1.62 ^e^	203.98 ± 4.22 ^b^
P10	221.43 ± 6.05 ^ef^	5.06 ± 0.16 ^abc^	226.49 ± 6.21 ^de^
P11	215.23 ± 1.23 ^e^	5.19 ± 0.07 ^abc^	220.42 ± 1.30 ^d^
Range	160.88–268.16	4.83–8.54	167.83–276.70
Average	216.37 ± 37.05	6.05 ± 1.23	222.42 ± 37.08

Note: Data are represented as mean of three different determinations ± standard deviation. Different letters in the same row indicate significant differences at *p* ≤ 0.05.

## Supplementary Materials

The followings are available online at https://www.mdpi.com/2304-8158/9/1/30/s1, Table S1: Origins and location of all seed samples used in this study, Table S2: Fatty acid composition and content in kernel and coat of the *P. ostii* seeds from 11 production areas, Table S3: The content of compounds detected in the seed kernel of the *P. ostii* from 11 production areas, Table S4: The APC index of *P. ostii* seeds from 11 production areas.
