# Supplementary material for: Fatty Acid Composition, Phytochemistry, Antioxidant Activity on Seed Coat and Kernel of Paeonia ostii from Main Geographic Production Areas
Source: Foods. 2019 Dec 28;9(1):30. doi: 10.3390/foods9010030 (PMC7022864; doi:10.3390/foods9010030)
Supplement: Supplementary file 1 [file foods-09-00030-s001.pdf]

Fatty acid composition, phytochemistry, antioxidant activity on seed coat and kernel of *Paeonia ostii* from main geographic production areas

Li-Ping Peng[a], Si-Qi Men[ab], Zheng-An Liu[a]*, Ning-Ning Tong[ab], Muhammad Imran[c], Qing-Yan Shu[a]*

[a]Key Laboratory of Plant Resources and Beijing Botanical Garden, Institute of Botany, the Chinese Academy of Sciences, Beijing 100093, China

[b]University of the Chinese Academy of Sciences, Beijing 100049, China

[c]State Key Laboratory of Plant Cell and Chromosome Engineering, Institute of Genetics and Developmental Biology, Chinese Academy of Sciences, Beijing, 100101, China

*** Correspondence:**

Dr. Zheng-An Liu (Email: liuzhengan@ibcas. ac.cn)

Dr. Qing-Yan Shu (Email: shuqy@ibcas.ac.cn)

Supplemental Tables

Table S1. Origins and location of all seed samples used in this study

Table S2. Fatty acid composition and content in kernel and coat of the *P. ostii* seeds from 11 production areas.

Table S3. The content of compounds detected in the seed kernel of the *P. ostii* from 11 production areas.

Table S4. The APC index of *P. ostii* seeds from 11 production areas.

Table S1. Origins and location of all seed samples used in this study

Code	Origin	Abbreviation	Longitude	Latitude
P1	Leshan city in Sichuan province	SCLS	103.7654	29.5533
P2	Dazhou city in Sichuan province	SCDZ	107.4679	31.2097
P3	Tangshan city in Hebei province	HBTS	118.1806	39.6315
P4	Heze city in Shandong province	SDHZ	115.4812	35.2347
P5	Jiaozuo city in Henan province	HNJZ	113.2420	35.2166
P6	Zaozhuang city in Shandong province	SDZZ	117.3220	34.8121
P7	Liaocheng city in Shandong province	SDLC	115.9855	36.4584
P8	Pingliang city in Gansuprovince	GSPL	106.6653	35.5441
P9	Luoyang city in Henan province	HNLY	112.4536	34.6232
P10	Gaoyi city in Hebei province	HBGY	114.61148	37.6159
P11	Bozhou city in Anhui province	AHBZ	115.7793	33.8460

Table S2. Fatty acid composition and content in kernel and coat of the *P. ostii* seeds from 11 production areas.

Code	Palmitic Acid (%)		Stearic Acid (%)		Oleic Acid (%)	
	Seed Coat	Seed Kernel	Seed Coat	Seed Kernel	Seed Coat	Seed Kernel
P1	6.37±0.31bc	3.94±0.25cd	1.79±0.18c	1.15±0.05cd	26.63±0.97a	34.88±0.53a
P2	4.59±0.43a	3.79±0.09bcd	1.09±0.16ab	0.95±0.23abc	31.84±1.19ab	35.44±1.25ab
P3	5.56±1.11ab	3.89±0.09bcd	1.32±0.16abc	0.91±0.10ab	31.62±2.8ab	36.45±0.74bcd
P4	5.65±0.7ab	3.64±0.11ab	1.45±0.27bc	1.07±0.04bcd	31.04±1.42b	36.36±0.21bcd
P5	6.13±1.07bc	3.69±0.19abc	1.52±0.23bc	1.07±0.10bcd	33.89±2.33cd	37.59±0.37de
P6	4.47±0.29a	3.79±0.17bcd	1.01±0.22ab	0.95±0.14abc	32.92±0.93ab	37.20±0.31cde
P7	6.10±0.87bc	3.98±0.08d	1.77±0.54c	1.12±0.14bcd	27.64±1.55a	36.22±0.19bc
P8	4.54±0.46a	3.86±0.11bcd	0.99±0.14ab	1.08±0.15bcd	32.68±0.85ab	38.10±0.77e
P9	4.62±0.34a	3.51±0.02a	0.88±0.15a	1.01±0.02bcd	36.39±0.15de	39.51±0.43f
P10	6.97±0.49cd	3.9±0.09bcd	1.84±0.21c	0.77±0.04a	38.81±0.09e	41.02±0.71g
P11	7.94±0.57d	3.71±0.1abc	2.70±0.47d	1.23±0.04d	25.78±1.72a	35.50±1.10ab
Range	4.47-7.94	3.51-3.98	0.88-2.70	0.77-1.23	25.78-38.81	34.88-41.02
Average	5.72±1.22	3.79±0.17	1.49±0.56	1.03±0.15	31.75±4.03	37.12±1.88

Code	Linoleic Acid (%)		α-Linolenic (%)		Unsaturated Fatty Acid (%)	
	Seed Coat	Seed Kernel	Seed Coat	Seed Kernel	Seed Coat	Seed Kernel
P1	13.26±0.64b	22.43±0.54f	51.92±0.99e	37.57±1.18bcd	91.83±0.43bc	94.89±0.23a
P2	16.50±0.11c	22.83±0.52f	45.96±0.57ab	36.97±1.41bc	94.31±0.6d	95.25±0.16abc
P3	13.34±0.88b	21.06±0.35d	48.14±3.13cd	37.67±1.14bcd	93.11±1.27cd	95.19±0.08abc
P4	15.81±1.24c	22.14±0.63ef	46.03±1.03ab	36.76±0.67bc	92.89±0.98bcd	95.27±0.16bc
P5	10.62±1.93a	18.74±0.66b	47.81±1.73cd	38.88±0.19d	92.34±1.31bc	95.22±0.28abc
P6	20.19±0.6d	24.63±0.28g	41.38±1.00a	33.41±0.19a	94.5±0.52d	95.25±0.25abc
P7	15.17±1.75c	21.58±0.30de	49.29±2.01d	37.06±0.32bc	92.11±1.4bc	94.88±0.22a
P8	13.20±0.44b	19.37±0.65bc	48.57±0.61cd	37.56±0.44bcd	94.46±0.6d	95.04±0.26ab
P9	12.93±1.01b	19.74±0.21c	45.16±0.48b	36.2±0.26b	94.49±0.49d	95.46±0.03c
P10	10.79±0.70a	17.27±0.52a	41.57±0.11a	37.01±0.31bc	91.18±0.68b	95.31±0.06bc
P11	11.56±0.42ab	21.48±0.14de	51.99±0.94e	38.04±1.12cd	89.34±1.04a	95.04±0.13ab
Range	10.62-20.19	17.27-24.63	41.38-51.99	33.41-38.88	89.34-94.50	94.88-95.46
Average	13.94±2.86	21.03±2.06	47.08±3.61	37.01±1.49	92.78±1.76	95.16±0.23

Note: Date represents mean of three different determinations±standard deviation. Different tiny letters in the same row indicate significant differences at $p \leq 0.05$

Table S3. The content of compounds detected in the seed kernel of the *P. ostii* from 11 production areas.

Code	Peak1	Peak 2	Peak 3	Peak 4	Peak 5	Peak 6	Peak 7	Peak 8	Peak 9
P1	0.72±0.01abc	0.00±0.00a	0.98±0.04a	3.90±0.15ab	1.94±0.08a	16.51±0.68cd	2.78±0.02d	1.31±0.01bcd	21.60±0.28c
P2	0.61±0.00ab	0.58±0.01bc	1.70±1.27ab	4.65±0.09b	1.43±0.02b	14.15±0.38de	0.35±0.01b	1.38±1.03cd	13.94±0.24ab
P3	0.97±0.05cd	0.96±0.04e	4.06±0.08cd	6.91±0.28c	0.92±0.01cde	41.16±1.33a	0.00±0.00a	1.91±0.09d	30.23±1.59d
P4	0.81±0.03abcd	3.50±0.11f	4.34±0.06cd	3.74±0.07ab	0.57±0.01f	20.42±0.84c	1.76±0.04bc	0.70±0.02ab	18.54±0.25bc
P5	0.93±0.03cbd	0.48±0.02b	3.94±0.13cd	3.26±0.22a	0.89±0.05def	11.93±0.62ef	1.92±0.02bc	0.75±0.01abc	14.18±0.52ab
P6	0.57±0.01a	0.59±0.01bc	4.35±0.05cd	3.08±0.01a	1.22±0.01bc	11.43±0.06ef	1.94±0.04bc	1.25±0.03bc	13.91±0.20ab
P7	0.75±0.03abcd	0.55±0.04bc	3.99±0.10cd	3.73±0.22ab	1.17±0.08bcd	18.83±0.70c	2.03±0.07bcd	1.08±0.05abc	15.42±0.54ab
P8	0.55±0.04a	0.81±0.02d	5.52±0.22d	3.94±0.16ab	0.80±0.02ef	31.34±0.11b	1.37±0.00b	0.56±0.02a	20.53±0.20c
P9	0.66±0.57abc	0.50±0.15c	3.07±2.38bc	3.15±1.79a	0.64±0.56ef	8.83±7.66f	1.69±1.47cd	0.58±0.51a	11.80±9.23a
P10	1.07±0.02d	0.61±0.04bc	4.70±0.07d	7.17±0.18c	1.13±0.05bcd	27.63±0.77b	2.46±0.07cd	1.03±0.05abc	21.83±0.07c
P11	0.76±0.00abcd	0.58±0.03c	4.17±0.03cd	3.32±0.09a	0.87±0.03def	13.14±0.26def	2.10±0.04bcd	0.65±0.02ab	14.56±0.38ab
Range	0.55-1.07	0.00-3.50	0.98-5.52	3.08-7.17	0.57-1.94	8.83-41.16	0.00-2.78	0.56-1.91	11.80-30.23
Average	0.76±0.22	0.83±0.89	3.71±1.45	4.26±1.48	1.05±0.39	19.58±9.95	1.67±0.89	1.02±0.50	17.87±5.71
Code	Peak 10	Peak 11	Peak 12	Peak 13	Peak 14	Peak 15	Peak 16	Peak 17	

P1	0.00±0.00a	1.72±0.11g	0.84±0.07a	1.07±0.02e	2.89±0.15f	1.48±0.05ab	4.39±0.27c	0.00±0.00a	
P2	0.00±0.00a	0.29±0.01b	0.82±0.03a	0.00±0.00a	0.70±0.05a	1.11±0.04a	1.88±0.13a	3.67±0.18d	
P3	4.93±0.289f	0.00±0.00a	3.07±4.04b	0.45±0.02b	1.13±0.10abcd	3.62±0.16d	3.64±0.30c	2.09±0.08b	cc
P4	0.21±0.01ab	1.17±0.02f	0.60±0.02a	0.52±0.01b	1.41±0.15bcd	1.49±0.07ab	6.96±0.27d	4.38±0.14f	
P5	0.14±0.13ab	0.69±0.04cd	0.50±0.03a	0.00±0.00a	0.90±0.03ab	1.41±0.03ab	1.52±0.02a	4.28±0.24ef	
P6	0.00±0.00a	0.85±0.05de	0.76±0.01a	0.75±0.02c	2.12±0.06e	2.88±0.06cd	4.17±0.07c	4.28±0.10ef	
P7	0.64±0.01c	1.02±0.04ef	0.81±0.02a	0.51±0.01b	1.59±0.03cde	2.42±0.11bc	4.58±0.21c	4.07±0.18e	
P8	1.31±0.13d	0.75±0.01cd	0.49±0.03a	0.5±0.01b	1.62±0.20cde	1.99±0.02abc	4.36±0.15c	4.19±0.11ef	
P9	0.32±0.28b	0.51±0.45bc	0.41±0.36a	0.45±0.40b	1.23±1.07abcd	2.64±2.12bcd	2.38±1.90a	2.82±0.06c	
P10	1.79±0.17e	0.83±0.04de	0.71±0.04a	0.00±0.00a	1.03±0.07abc	3.68±0.15d	2.51±0.21ab	1.99±0.11b	
P11	0.00±0.00a	0.92±0.03def	0.50±0.03a	0.62±0.01bc	1.71±0.19de	2.24±0.06abc	3.52±0.02bc	2.14±0.05b	
Range	0.00-4.93	0.00-1.72	0.41-3.07	0.00-1.07	0.70-2.89	1.11-3.68	1.52-6.96	0.00-4.38	
Average	0.85±1.44	0.80±0.45	0.87±1.25	0.44±0.34	1.48±0.66	2.27±1.01	3.63±1.57	3.08±1.37	

Note: Date represents mean of three different determinations±standard deviation. Different tiny letters in the same row indicate significant differences at $p \leq 0.05$

Table S4. The APC index of *P. ostii* seeds from 11 production areas.

Seed Coat	APC Index（%）			APC Comprehensive Index（%）	Rank
	DPPH	FRAP	ABTS		
P1	71.62	78.46	89.09	79.72	3
P2	64.70	70.88	78.66	71.41	6
P3	69.87	83.90	89.60	81.12	1
P4	64.41	67.60	72.58	68.20	9
P5	70.58	72.00	74.86	72.48	5
P6	68.46	71.07	78.75	72.76	4
P7	66.20	71.91	75.73	71.28	7
P8	67.91	69.76	70.47	69.38	8
P9	76.49	76.59	87.16	80.08	2
P10	66.51	69.76	65.64	67.30	10
P11	61.56	68.07	69.38	66.34	11

Seed Kernel	APC Index（%）			APC Comprehensive Index（%）	Rank
	DPPH	FRAP	ABTS		
P1	74.07	62.26	92.98	76.44	9
P2	66.67	66.04	78.07	70.26	11
P3	87.04	73.58	93.86	84.83	5
P4	79.63	79.25	95.61	84.83	4
P5	75.93	66.04	73.68	71.88	10
P6	74.07	69.81	92.11	78.66	8
P7	75.93	71.70	91.23	79.62	7
P8	90.74	84.91	93.86	89.84	2
P9	85.19	81.13	90.35	85.56	3
P10	100.00	100.00	100.00	100.00	1
P11	83.33	77.36	84.21	81.63	6

Note: Lowercase letters indicate significance at $p \leq 0.05$.
